# Association between dietary patterns and metabolic syndrome in a sample of portuguese adults

**DOI:** 10.1186/1475-2891-11-64

**Published:** 2012-09-03

**Authors:** Maria João Fonseca, Rita Gaio, Carla Lopes, Ana Cristina Santos

**Affiliations:** 1 Institute of Public Health, University of Porto, Rua das Taipas nº 135, Porto, 4050-600, Portugal; 2 Department of Mathematics, Science Faculty, University of Porto, Portugal. Centre for Mathematics, University of Porto, Porto, Portugal (CMUP; 3 Department of Clinical Epidemiology, Predictive Medicine and Public Health and Cardiovascular Research & Development Unit, University of Porto Medical School, Porto, Portugal

**Keywords:** *a posteriori* dietary patterns, Finite mixture model, Metabolic syndrome, Portugal

## Abstract

**Background:**

There is scarce evidence regarding the association between diet and metabolic syndrome (MetS) in Portuguese population. We aim to evaluate the association between *a posteriori* dietary patterns (DPs) and MetS and its features.

**Methods:**

Using random digit dialing, a sample of 2167 adults was selected between 1999 and 2003, in Porto. During a face-to-face interview, a questionnaire was applied, anthropometric measures were taken, blood pressure measured and a fasting blood sample collected. Diet was assessed using a validated food frequency questionnaire, and four DPs were identified in each sex by multivariate finite mixture models.

**Results:**

After adjustment for age and daily energy intake, comparing to the “healthy” DP, women with the “low fruit and vegetables” DP had a higher odds of high waist circumference (OR = 1.88 95% CI 1.17-3.01) and low HDL-cholesterol (OR = 1.78 95% IC 1.12-2.82) and women in the “red meat and alcohol” DP had higher odds of high waist circumference (OR = 1.45 95% CI 1.01-2.07) and of MetS (OR = 1.57 95% CI 1.07-2.29); men with the “fish” DP had a higher odds of high triglycerides (OR = 1.57 95% CI 1.05-2.35). After further adjustments (education, physical activity, smoking, alcohol drinking, BMI, and menopausal status) no significant associations remained.

**Conclusions:**

Four distinct DPs were identified in a community sample of Portuguese adults and there was no association with the prevalence of MetS.

## Background

Five features have been established for metabolic syndrome (MetS): central obesity, high fasting glucose, triglycerides and blood pressure, and low HDL-cholesterol [1-3]. Although the prevalence of MetS differs according to the used criteria, both its prevalence and incidence are rising [4,5], and, in 2007, within an urban Portuguese sample, the prevalence estimated was 37.2% [6].

There is no uniform consensus regarding which dietary approach is most efficacious in preventing and managing the MetS and its features [7]. The inconsistency of findings may be partly explained by the traditional single-nutrient approach, commonly used in nutritional epidemiological research. The limitation more often pointed to this kind of approach is the difficulty in detecting small effects from single nutrients. Also, it is uncertain whether the observed association actually reports the specific effect of the nutrient intake, or if it acts as an indicator of an overall lifestyle [8].

An alternative approach that obviates this limitation is the establishment of dietary patterns (DPs), which can be established, *a priori* or *a posterior*. Several DP analyses have relied on *a priori* approaches [9,10], and are based on the assumption of protective or harmful health effects of food components; on the other hand this approach does not acknowledge that clustering of food components can vary across populations, reflecting cultural traditions. Another approach has been proposed: the *a posteriori* construction of DPs [11], which takes into consideration the real eating behaviours of the population, without making any assumption of protective or harmful health effects. These DPs are constructed based on statistical models, such as factor, cluster or principal component analysis.

Most of the previous studies have focused on the effect of diet on each of the individual features of MetS [12-17]. It was established that the reduction in total energy intake was the most important determinant for lower body weight [12] and diets with high fruit, vegetables and low-fat dairy products intake, along with reduced sodium intake, were associated to decreased blood pressure [14]. Regarding the management of MetS as a clinical entity, the recommended diet includes a total fat intake within 25–35%, reduction of saturated fat (<7%), trans fat, cholesterol (<200 mg/d), and simple sugars, also most dietary fat should be unsaturated [3].

The aim of this study aim was to evaluate the association between *a posteriori* DPs and the prevalence of MetS and its components in the adult population of Porto, Portugal.

## Participants and methods

### Study design and participants

This study is a cross-sectional analysis of data from EPIPorto cohort, which has been previously described [6,18]. The recruitment started in 1999 and lasted until 2003. Participants were non-institutionalised inhabitants of Porto, Portugal, who visit the Department of Hygiene and Epidemiology of University of Porto Medical School for an interview and examination. The proportion of participation of the EPIPorto cohort at assembling was 70% [18].

For this study, 2485 participants met the inclusion criteria. Mini-mental state examination was used for the evaluation of cognitive impairment in individuals aged over 64 years [19], and those who scored less than 24 were considered as unreliable to provide information and therefore excluded (n = 19). Because of missing information, 299 participants were excluded, remaining for analysis 2167 participants (1330 women and 837 men).

All participants gave their written informed consent and the local institutional ethics committee, as well as, the National Individual Data Protection Commission, approved the EPIPorto protocol.

### Data collection

During the visit to the Department, trained staff conducted a structured questionnaire, comprising self-reported information on socio-demographic and behavioural characteristics, and personal and family medical history.

Age and education were recorded as completed years of age and schooling. Physical activity was assessed by a validated questionnaire exploring all professional, domestic and leisure time activities over the past 12 months [20]. Information on current and previous smoking status was also self-reported.

Dietary intake was assessed using a validated semi-quantitative Food Frequency Questionnaire (FFQ) [21,22], comprising 82 items, reporting to the previous year. Each subject chose the average frequency of consumption for each food-item among nine categories, ranging from “never or less than once a month” to “6 or more times a day”; subjects also reported the average portion consumed (lower, equal or higher than the average portion size) and the seasonal variation of consumption. Food consumption was converted into total energy intake and nutrients using the software Food Processor Plus® (ESHA Research, Salem-Oregon, 1997), adapted to traditional Portuguese foods.

Anthropometric measurements were performed with subjects in light clothing and barefoot, after 12 hours fasting, and under standard procedures. Body weight was measured to the nearest 0.1 kilogram using a digital scale (SECA®, Columbia, USA), and height to the nearest centimetre using a wall stadiometer (SECA®, Hamburg, Germany). BMI was calculated. Waist circumference (WC) was ascertained as the midway between the lower limit of the rib cage and the iliac crest, and hip circumference on the maximum circumference over the femoral trochanters; both were measured to the nearest centimetre.

Blood pressure was measured on a single occasion using a standard mercury sphygmomanometer with the cuff on the right upper arm. Two blood pressure readings were taken with the participant resting for 10 minutes, and the mean of the two readings was calculated. If the difference between these two readings was more than 5 mmHg for systolic or diastolic blood pressure, a third measurement was taken and the mean of the two closest values was considered.

Blood was sampled after a 12-hour overnight fast to measure glucose, total cholesterol, HDL-cholesterol, and triglycerides.

### Definition of MetS

MetS was defined according to the most recent proposed definition and was considered present when at least three of the following characteristics were present: WC ≥102 cm in men and ≥88 cm in women; triglycerides ≥150 mg/dl and/or lipid lowering medication; HDL-cholesterol <40 mg/dl in men and <50 mg/dl in women and/or lipid lowering medication; blood pressure ≥130/85 mmHg and/or antihypertensive medication; and fasting glucose ≥100 mg/dl and/or antidiabetic medication [1].

### Statistical analysis

#### Dietary pattern analysis

The 82 food items of the FFQ were aggregated into 14 groups according to nutritional similarities. Since significant sex differences were found among food-group distributions (p < 0.001), DPs were identified separately for men and women, using multivariate finite mixture models [23].

Food-groups with a percentage of non-consumption higher than 45% (alcohol in women and soft drinks in both genders) were dichotomized into consumption and non-consumption, to avoid high frequencies of zero consumption, and thus problems in model fitting. Besides alcohol in women and soft drinks in both genders, the remaining food-groups presented non-consumptions <15%. The variables that were not dichotomized were log-transformed, after adding 1(g) to avoid zero values.

Screening of outliers was done by the sigma gap method for right skewed distributions. Twenty-four men and 22 women were identified as outliers and removed for the definition of patterns.

The multivariate dependent variable represented the 14 food-group consumptions. Differences in food consumption with age, as well as high correlations between food consumption and total energy intake, were detected. The simultaneous linear regressions of food group consumptions and multinomial regressions of pattern prevalence were therefore taken on age group and total energy intake. For the model structure, variances of the continuous food group intakes were allowed to vary within and between patterns.

The final number of clusters (n = 4, for each sex) was established by the cessation of the monotonically decrease of the Bayesian Information Criterion. The mean (and standard deviation) of the individual *a posteriori* probabilities (probabilities predicting the cluster membership) were 89% (0.14) in women and 92% (0.13) in men.

#### Other statistical analysis

Proportions were compared using the chi-square test, and means were compared using ANOVA or Kruskal-Wallis test, as appropriated.

To estimate the magnitude of the association between DPs and MetS and its individual components, crude and adjusted odds ratio (OR) and their respective 95% confidence intervals (95% CI) were computed using unconditional logistic regression models, according to gender. OR were adjusted for age, daily energy intake, education, BMI, physical activity, smoking, alcohol consumption, and, in women, for their menopausal status. Models were established based on the confounding effect of these variables.

Statistical analyses for DPs identification were mainly conducted with the package flexmix from the software R, version 2.7.1, (R Foundation for Statistical Computing, Austria, 2008). The remaining analyses were performed with SPSS, version 18.0 (SPSS Inc., Chicago, IL, USA).

## Results

Four distinct groups were identified regarding food intake, separately for men and women. These 8 DPs were already identified and described, in previous studies [24-26]. The distribution of women by the identified DPs was: 23.8% (317) in cluster 1 (“healthy”), 8.9% (119) in cluster 2 (“low fruit and vegetables”), 19.7% (262) in cluster 3 (”red meat and alcohol”) and 47.5% (632) in cluster 4 (“in transition to fast-food”). A total of 21.9% (183) men were grouped in cluster 1 (“healthy”), 42.7% (357) in cluster 2 (“fish”), 22.0% (184) in cluster 3 (“red meat and alcohol”), and 13.5% (113) in cluster 4 (“intermediate”).

Mean consumption of nutrient intake in each cluster are shown in Table 1. Women in the “healthy” DP had the highest consumption of vegetables, fruits and dairy products, and the lowest consumption of red meat, fast-food and soft drinks; therefore, these women had the lowest intake of fat, cholesterol, omega 6 fatty acids and ethanol and the highest of carbohydrates, dietary fibre and some minerals and vitamins. The “low fruit and vegetables” DP was characterized by the lowest intake of most food-groups, particularly fruit and vegetables; so, these women had the lowest energy intake, as well as, the lowest intake of most of the minerals and vitamins. The “red meat and alcohol” DP was the one with the highest consumption of red meat and alcoholic beverages and the lowest consumption of dairy products and vegetable soup; these women had the lowest consumption of protein, calcium and vitamin D. The “in transition to fast-food” DP was characterized by the highest intake of white meat, sweets and fast-foods and the second highest intakes of red meat, vegetables and dairy products; thus, these women had the highest caloric intake, and the highest consumption of protein, total fat, saturated and monounsaturated fat, cholesterol, omega 3 and 6 fatty acids, potassium and vitamin E and had the lowest intake of carbohydrates (Table 1). Men in the “healthy” DP had the highest consumption of vegetable soup, fruits, dairy products and cereals, and the lowest of red meat, fast-food and alcoholic beverages; as a result, these men had the highest intake of carbohydrates, proteins, and polyunsaturated and monounsaturated fat and the lowest of saturated fat and ethanol; these men also had the highest intake of dietary fibre and of most of the minerals and vitamins. The “fish” DP was characterized by the highest intake of fish and vegetables and also the highest energy intake, monounsaturated fat, omega 3 fatty acids and potassium. The “red meat and alcohol” DP had the highest consumption of red meat, alcohol and fast-food, and the lowest of fruits, vegetable soup, dairy products and cereals; thus, this DP presented the highest intake of saturated, polyunsaturated and total fat, cholesterol, omega 6 fatty acids and ethanol, and the lowest intake of carbohydrates, dietary fibre and all minerals and vitamins analyzed with the exception of potassium. The “intermediate” DP had an intermediate consumption of most food-groups (except white meat, sweets and soft-drinks); these men had the lowest energy intake, as well as the lowest intake of protein and fat (total, polyunsaturated and monounsaturated), cholesterol, omega 3 and 6 fatty acids, and potassium (Table 1).

**Table 1:** Mean nutrient intake in each identified cluster in women and men

		**Women**			**Men**			
		**Dietary pattern [mean(sd)]**			**Dietary pattern [mean(sd)]**			
	**“healthy"**	**“low fruit and vegetables”**	**“red meat and alcohol”**	**“In transition to fast-food”**	**“healthy”**	**“fish”**	**“red meat and alcohol”**	**“intermediate”**
	**(n = 317)**	**(n = 119)**	**(n = 262)**	**(n = 632)**	**(n = 183)**	**(n = 357)**	**(n = 184)**	**(n = 113)**
Calories (kcal/day)	1887 (439)	1827 (623)	1944 (481)	2189 (533)	2438 (622)	2587 (623)	2527 (610)	2334 (686)
Carbohydrates (% kcal)	54.2 (6.4)	49.9 (9.5)	50.9 (6.4)	49.5 (5.6)	52.3 (5.8)	45.1 (5.5)	41.4 (6.6)	50.0 (8.2)
Protein (% kcal)	18.3 (2.4)	18.3 (3.9)	18.1 (2.6)	18.7 (2.5)	18.0 (2.4)	17.4 (3.4)	17.5 (2.6)	16.9 (3.1)
Total fat (% kcal)	27.5 (4.6)	29.8 (6.0)	29.3 (4.6)	31.2 (4.2)	28.3 (5.2)	28.6 (4.4)	29.0 (5.3)	27.8 (6.0)
Saturated fat (% kcal)	8.2 (2.1)	9.4 (3.0)	9.0 (2.0)	9.9 (1.9)	8.4 (2.3)	8.9 (2.0)	9.1 (2.3)	8.7 (2.7)
Polyunsat. fat (% kcal)	4.4 (0.9)	4.9 (1.1)	5.1 (1.0)	5.0 (0.8)	4.9 (1.0)	4.7 (0.8)	4.9 (1.0)	4.7 (1.1)
Monounsat. fat (% kcal)	12.4 (2.7)	12.9 (3.3)	12.5 (2.5)	13.6 (2.3)	12.5 (2.5)	12.5 (2.2)	12.4 (2.4)	12.0 (2.7)
Cholesterol (mg/day)	223.3 (80.7)	254.5 (115.8)	277.7 (99.9)	325.9 (108.1)	317.6 (128.9)	351.4 (111.8)	360.2 (109.6)	296.6 (138.5)
Omega 3 (g/day)	1.18 (0.39)	1.08 (0.40)	1.20 (0.37)	1.37 (0.44)	1.39 (0.44)	1.49 (0.45)	1.40 (0.43)	1.27 (0.47)
Omega 6 (g/day)	6.7 (2.2)	7.2 (3.1)	8.0 (2.7)	8.8 (3.0)	9.5 (3.7)	9.7 (3.4)	10.2 (3.9)	8.7 (3.9)
Ethanol (% kcal)	2.0 (4.0)	3.4 (5.6)	3.0 (4.8)	2.1 (3.2)	2.8 (4.8)	10.0 (6.4)	12.7 (9.3)	6.3 (8.7)
Dietary fibre (g/day)	25.9 (8.9)	18.2 (8.1)	20.6 (6.6)	23.3 (7.8)	28.3 (9.3)	24.7 (7.4)	19.2 (6.7)	22.1 (8.5)
Calcium (mg/day)	1030.0 (389.9)	789.4 (470.3)	664.0 (319.7)	972.2 (353.8)	1001.0 (376.5)	917.4 (304.6)	707.2 (364.3)	817.0 (435.6)
Potassium (mg/day)	3650.7 (991.3)	2962.8 (1006.9)	3111.7 (783.7)	3762.4 (941.7)	4082.4 (985.1)	4157.3 (933.6)	3630.0 (840.5)	3541.3 (1006.1)
Vitamin C (mg/day)	148.2 (66.2)	113.8 (76.7)	120.0 (63.2)	139.1 (61.6)	144.0 (62.6)	132.0 (53.9)	94.4 (40.8)	112.0 (64.8)
Vitamin E (mg/day)	7.9 (2.4)	6.6 (2.6)	7.0 (2.2)	8.3 (2.4)	9.1 (2.7)	8.6 (2.4)	7.2 (2.2)	7.4 (2.6)
Vitamin A (mg/day)	1833.8 (857.8)	1132.3 (651.4)	1396.3 (963.9)	1688.0 (832.4)	1725.9 (769.8)	1614.0 (731.6)	1131.3 (675.5)	1308.0 (880.1)
Vitamin D (mg/day)	4.3 (2.9)	3.5 (2.6)	3.3 (1.9)	4.2 (2.5)	4.4 (2.6)	4.1 (1.9)	3.5 (1.7)	7.4 (2.6)
Folate (mcg/day)	314.4 (128.4)	234.8 (104.8)	249.7 (91.6)	297.1 (108.4)	334.6 (111.1)	308.4 (102.7)	260.6 (90.5)	271.2 (112.5)

Socio-demographic, lifestyle and anthropometric characteristics, according to DPs, are shown in Table 2. In women, the “healthy” DP was the one with the highest mean age [58 years (SD 15)], the lowest proportion of current smoking (9.1%) and drinking (36.3%), and the highest proportion of women practicing regular physical activity (42.6%). The “low fruit and vegetables” DP had the highest proportion of current smoking (29.4%) and drinking (47.9%). Women in “red meat and alcohol” DP had the lowest education level [median 4 years of school (IQR 7.0)] and the lowest proportion of women practicing regular physical activity (22.1%). In the “in transition to fast-food” cluster were the youngest women [mean 48 years (SD 14)]. In men, the “healthy” DP had the lowest proportion of smokers (23.0%) and alcohol drinkers (48.1%), and the highest proportion of men practicing regular physical activity (54.1%). In the “fish” DP there was the highest proportion of current drinkers (98.6%). Men with the “red meat and alcohol” DP were the youngest [mean 48 years old (SD 14)], this cluster had the highest proportion of current smokers (46.2%) and the lowest of men practicing regular physical activity (31.0%) (Table 2).

**Table 2:** Socio-demographic, lifestyle and anthropometric characteristics, according to dietary patterns

	**Women**					**Men**				
	**“healthy"**	**“low fruit and vegetables”**	**“red meat and alcohol”**	**“In transition to fast-food”**	**p**	**“healthy”**	**“fish”**	**“red meat and alcohol”**	**“intermediate”**	**p**
*mean (SD)*										
Age, years	58 (15)^a,b,d^	51 (15)^a^	52 (14)^bc^	48 (14)^c,d^	p < 0.001	55 (19)^a^	55 (13)^b^	48 (14)^a,b,c^	54 (17)^c^	p < 0.001
Education, years*	6 (8)	9 (11)	4 (7)	9 (11)	p < 0.001	9 (11)	9 (9)	9 (8)	9 (8)	p = 0.426
Body mass index, kg/m^2^	27.1 (4.5)	27.4 (5.4)	27.8 (5.8)^a^	26.6 (5.0)^a^	p = 0.018	25.7 (3.9)^a,b^	26.6 (3.5)^a^	26.8 (4.1)^b^	25.7 (4.3)	p = 0.006
Waist circumference, cm	86.6 (11.9)^b^	87.2 (14.5)	88.0 (13.7)^a^	84.2 (12.5)^a,b^	p < 0.001	91.0 (10.9)^a^	93.8 (10.1)^a^	93.6 (10.6)	91.7 (11.7)	p = 0.015
Waist/Hip	0.85 (0.08)^b^	0.85 (0.08)	0.86 (0.08)^a^	0.83 (0.08)^a,b^	p < 0.001	0.91 (0.07)^a,b^	0.94 (0.09)^a^	0.94 (0.06)^b^	0.93 (0.08)	p = 0.001
Systolic blood pressure, mmHg	139 (26)^c^	138 (26)^a^	136 (26)^b^	130 (24)^a,b,c^	p < 0.001	135 (21)	136 (20)	135 (22)	138 (21)	p = 0.555
Diastolic blood pressure, mmHg	83 (11)	85 (13)^a^	83 (13)	81 (12)^a^	p = 0.002	82 (11)	83 (11)	84 (11)	83 (10)	p = 0.219
Total cholesterol, g/L	2.21 (0.50)	2.21 (0.48)	2.21 (0.49)	2.15 (0.42)	p = 0.114	2.00 (0.47)^a,b^	2.20 (0.46)^a^	2.22 (0.47)^b^	2.13 (0.44)	p < 0.001
HDL cholesterol, g/L	0.61 (0.13)	0.60 (0.18)	0.59 (0.14)	0.60 (0.15)	p = 0.372	0.50 (0.14)	0.50 (0.13)	0.51 (0.13)	0.49 (0.13)	p = 0.683
LDL cholesterol, g/L	1.38 (0.44)	1.39 (0.42)	1.38 (0.47)	1.34 (0.39)	p = 0.248	1.28 (0.42)^a,b^	1.42 (0.41)^a^	1.43 (0.41)^b^	1.37 (0.40)	p = 0.001
Fasting glucose, g/L*	0.89 (0.14)	0.89 (0.16)	0.89 (0.17)	0.87 (0.14)	p = 0.106	0.91 (0.17)	0.93 (0.15)	0.92 (0.17)	0.93 (0.14)	p = 0.335
Triglycerides, g/L*	0.97 (0.67)	0.91 (0.65)	0.98 (0.67)	0.87 (0.58)	p = 0.009	0.98 (0.70)	1.16 (0.88)	1.19 (0.97)	1.03 (0.99)	p < 0.001
Physical activity, mets*minute/day*	34.4 (5.0)	34.2 (6.6)	34.5 (6.7)	35.0 (5.6)	p = 0.010	34.3 (5.6)	34.3 (5.7)	34.1 (10.1)	33.5 (5.9)	p = 0.031
BDI*	8.0 (10.0)	10.0 (14.0)	8.0 (10.0)	7.0 (9.0)	p = 0.011	5.5 (6.0)	5.0 (7.0)	5.0 (7.0)	7.0 (9.0)	p = 0.108
*n (%)*										
Current smoking	29 (9.1)	35 (29.4)	40 (15.3)	125 (19.8)	p < 0.001	42 (23.0)	106 (29.7)	85 (46.2)	51 (45.1)	p = 0.001
Current drinking	115 (36.3)	57 (47.9)	125 (47.7)	301 (47.6)	p = 0.005	88 (48.1)	352 (98.6)	178 (96.7)	60 (53.1)	p = 0.335
Regular physical activity	135 (42.6)	37 (31.1)	58 (22.1)	194 (30.7)	p < 0.001	99 (54.1)	148 (41.5)	57 (31.0)	47 (41.6)	p < 0.001
Marital status										
Married	189 (59.6)	59 (49.6)	181 (69.1)	391 (61.9)	p < 0.001	141 (77.0)	314 (88.0)	146 (79.3)	81 (71.7)	p < 0.001
Single	38 (12.0)	28 (23.5)	34 (13.0)	116 (18.4)			29 (15.8)	18 (5.0)	25 (13.6)	17 (15.0)
Widower	71 (22.4)	20 (16.8)	35 (13.4)	61 (9.7)			7 (3.8)	10 (2.8)	7 (3.8)	5 (4.4)
Divorced	19 (6.0)	12 (10.1)	12 (4.6)	64 (10.1)			6 (3.3)	15 (4.2)	6 (3.3)	10 (8.8)
Menopausal	223 (70.6)	72 (61.5)	145 (55.3)	283 (44.8)	p < 0.001	-	-	-		

BDI - Beck Depression Inventory.

a,b,c – groups between which there were significant associations.

* Due to the non normal distribution, the median and the interquartile range (IQR) are presented.

The overall prevalence of MetS was 27.6% (28.9% in women; 25.4% in men). Women within the “in transition to fast-food” cluster had the lowest prevalence of MetS and all its individual components. Men in the “healthy” pattern had the lowest prevalence of MetS, central obesity, high triglycerides and high fasting glucose levels. The severity of MetS, measured by the number of MetS features present, varied across the DPs, both in men (p = 0.036) and women (p = 0.002). The DP with the highest proportion of women with none of the features of MetS was the “in transition to fast-food” (29.9%), and the “red meat and alcohol” (3.8%) and the “healthy” DP (3.8%) were those with the highest proportion of women with the 5 MetS individual features. In men, the “red meat and alcohol” pattern was the one with the highest proportion of men with none of the features (21.2%) and the “intermediate” cluster was the one with the highest proportion of men with the 5 individual features (2.7%) (Table 3).

**Table 3:** Prevalence of MetS and its individual features, according to the four DPs

	**Women**					**Men**				
	**“healthy"**	**“low fruit and vegetables”**	**“red meat and alcohol”**	**“In transition to fast-food”**	**P**	**“healthy”**	**“fish”**	**“red meat and alcohol”**	**“intermediate”**	**p**
*n (%)*										
High waist circumference	140 (44.3)	57 (49.1)	115 (43.9)	229 (36.6)	0.014	24 (13.3)	63 (17.8)	32 (17.4)	23 (20.4)	0.424
High triglycerides/ medication	71 (22.6)	23 (19.5)	53 (20.5)	106 (16.9)	0.193	46 (25.3)	122 (34.4)	61 (33.2)	34 (30.4)	0.178
Low HDL cholesterol/ medication	88 (27.9)	43 (36.4)	74 (28.8)	172 (27.5)	0.264	48 (26.8)	84 (23.9)	42 (23.3)	37 (33.3)	0.203
High blood pressure/ medication	221 (72.0)	75 (69.4)	164 (65.3)	337 (55.7)	<0.001	122 (70.9)	245 (70.6)	118 (66.7)	86 (78.9)	0.177
High glucose/ medication	71 (22.7)	21 (18.3)	59 (23.0)	98 (15.7)	0.020	48 (26.8)	106 (30.1)	51 (28.3)	30 (26.8)	0.835
MetS	107 (33.8)	40 (33.6)	90 (34.4)	148 (23.4)	<0.001	43 (23.5)	88 (24.6)	46 (25.0)	36 (31.9)	0.399
Number of MetS features										
0	56 (17.7)	24 (20.2)	59 (22.5)	189 (29.9)		36 (19.7)	52 (14.6)	39 (21.2)	21 (18.6)	
1	92 (29.0)	33 (27.7)	71 (27.1)	172 (27.2)		69 (37.7)	109 (30.5)	53 (28.8)	29 (25.7)	
2	62 (19.6)	22 (18.5)	42 (16.0)	123 (19.5)		35 (19.1)	108 (30.3)	46 (25.0)	27 (23.9)	
3	65 (20.5)	21 (17.6)	60 (22.9)	88 (13.9)		26 (14.2)	58 (16.2)	25 (13.6)	20 (17.7)	
4	30 (9.5)	16 (13.4)	20 (7.6)	40 (6.3)		14 (7.7)	29 (8.1)	21 (11.4)	13 (11.5)	
5	12 (3.8)	3 (2.5)	10 (3.8)	20 (3.2)	0.002	3 (1.6)	1 (0.3)	0 (0.0)	3 (2.7)	0.036

After adjustment for age and daily energy intake (model 1), no significant associations were observed in women belonging to the “in transition to fast-food” DP and MetS or its individual features. However, the “low fruit and vegetables” and the “red meat and alcohol” DPs were associated with a higher WC when compared to the “healthy” DP (OR = 1.88 95%CI: 1.17-3.01 and OR = 1.45 95%CI: 1.01-2.07, respectively). The “low fruit and vegetables” DP was also associated with low HDL-cholesterol (OR = 1.78 95%CI: 1.12-2.82), while the “red meat and alcohol” presented the highest prevalence of MetS (OR = 1.57, 95%CI: 1.07-2.29). In men, an association between “fish” DP and high triglycerides (OR = 1.57 95%CI: 1.05-2.35) was observed (model 1). After additional adjustment for age, daily energy intake, education, physical activity, smoking, alcohol drinking, and BMI, no significant associations remained between DP and MetS or its individual features (Table 4).

**Table 4:** Association between MetS and each of its features and the four identified DP

		**Women**				**Men**			
		**“healthy”**	**“low fruit and vegetables"**	**”red meat and alcohol”**	**“In transition to fast-food”**	**“healthy”**	**“fish”**	**“red meat and alcohol”**	**“intermediate”**
High waist circumference	Model 1	1	1.88 (1.17-3.01)*	1.45 (1.01-2.07)*	1.20 (0.88-1.65)	1	1.50 (0.89-2.52)	1.80 (0.99-3.28)	1.74 (0.92-3.30)
	Model 2	1	1.95 (0.94-4.06)	1.12 (0.64-1.96)	1.08 (0.67-1.73)	1	1.10 (0.46-2.65)	1.51 (0.55-4.20)	2.78 (0.96-8.01)
High triglycerides/ medication	Model 1	1	1.05 (0.61-1.80)	1.13 (0.75-1.72)	1.03 (0.72-1.49)	1	1.57 (1.05-2.35)*	1.50 (0.95-2.38)	1.28 (0.75-2.15)
	Model 2	1	0.82 (0.46-1.46)	0.97 (0.62-1.51)	1.00 (0.68-1.47)	1	1.29 (0.78-2.14)	1.10 (0.63-1.94)	1.07 (0.60-1.88)
Low HDL cholesterol/ medication	Model 1	1	1.78 (1.12-2.82)*	1.23 (0.85-1.79)	1.27 (0.92-1.76)	1	0.90 (0.59-1.36)	0.85 (0.52-1.38)	1.32 (0.78-2.21)
	Model 2	1	1.47 (0.90-2.41)	1.13 (0.76-1.68)	1.30 (0.92-1.82)	1	0.95 (0.57-1.57)	0.88 (0.49-1.56)	1.26 (0.73-2.18)
High blood pressure/ medication	Model 1	1	1.52 (0.84-2.74)	1.19 (0.77-1.83)	0.97 (0.67-1.40)	1	0.88 (0.56-1.39)	1.17 (0.70-1.95)	1.63 (0.86-3.10)
	Model 2	1	1.62 (0.84-3.12)	0.98 (0.62-1.56)	0.94 (0.64-1.39)	1	0.59 (0.32-1.08)	0.76 (0.39-1.46)	1.77 (0.88-3.59)
High glucose/ medication	Model 1	1	1.01 (0.58-1.78)	1.39 (0.92-2.10)	1.01 (0.70-1.47)	1	1.24 (0.82-1.87)	1.32 (0.82-2.13)	1.00 (0.58-1.72)
	Model 2	1	0.77 (0.42-1.43)	1.03 (0.66-1.63)	0.92 (0.62-1.37)	1	1.06 (0.64-1.75)	1.05 (0.60-1.85)	0.96 (0.54-1.71)
**MetS**	Model 1	1	1.48 (0.91-2.42)	1.57 (1.07-2.29)*	1.08 (0.77-1.52)	1	1.13 (0.74-1.74)	1.33 (0.81-2.19)	1.53 (0.90-2.62)
	Model 2	1	1.06 (0.59-1.91)	1.19 (0.76-1.87)	0.97 (0.65-1.44)	1	0.84 (0.48-1.45)	0.91 (0.49-1.69)	1.52 (0.83-2.78)

Model 1 OR adjusted for age and daily energy intake.

Model 2 OR adjusted for age, daily energy intake, education, physical activity, smoking, alcohol drinking, BMI, and menopausal status.

* p < 0.05.

## Discussion

We identified four distinct DPs, separately for men (“healthy”, “fish”, “red meat and alcohol” and “intermediate”) and women (“healthy”, “low fruit and vegetables”, “red meat and alcohol” and “in transition to fast-food”). The association between DPs and socio-demographic, lifestyle and anthropometric characteristics suggested that food choices are part of a larger pattern of health behaviours. For example, individuals clustered in the “healthy” DP were more often non-smokers, non-drinkers and physically active, both in men and women. The main objective of this study was to clarify the association between food choices in a Mediterranean country and the occurrence of MetS and its individual features. Food choices were evaluated considering *a posterior* defined DPs using finite mixture models, which recent research suggested has some advantages over the previous methods [23]. In our sample, no association was observed between different DPs and the occurrence of MetS, after adjustment for the relevant confounders of this association.

A study performed in Greece, a country with similar food habits, concluded that a DP characterized by the consumption of cereals, fish, legumes, vegetables, and fruits, like our “healthy” DP, was associated with a lower risk of MetS (OR = 0.87 95% CI 0.79-0.97); also, DPs characterized by the intake of potatoes and meat and by alcohol intake were positively associated with MetS (OR = 1.13 95% CI 1.05-1.21 and OR = 1.26 95% CI 1.21-1.33, respectively). A systematic review [27] on the association between DPs and MetS came up with some conclusions: DPs characterized by high fruit and vegetables intake were often associated with lower prevalence of MetS; DPs with high meat intake were associated with features of MetS, especially with impaired glucose tolerance; high dairy intake was associated with reduced risk of MetS features but there were some inconsistency regarding risk of obesity; minimally processed cereals also seemed to be associated with reduced risk of MetS, while highly processed cereals with high glycemic index were associated with higher risk. The major conclusion of this review was that the overall quality of the diet appeared to protect against MetS, and no individual dietary component could be considered totally responsible for this association [27]. This conclusion supports our methodology of using DPs to evaluate diet.

Nevertheless, other studies have observed similar results to ours [28,29]. In the Bogalusa Heart Study, in a sample of 995 young USA adults (19–39 years old), the described *a posteriori* DPs (Western and Prudent) were not associated with the overall prevalence of MetS [28]. Also, in a sample of 944 Korean adolescents (10–19 years old) no association was observed between the *a posteriori* DPs (Korean traditional, Western and Modified DPs) and the MetS [29].

One possible explanation for the absence of association between the different DPs and the occurrence of MetS could be the fact that no group of people clustered in a way that their food intake, lifestyle habits and socio-demographic characteristics were the ideal for the prevention and management of MetS and its features.

### Limitations and strengths

The cross-sectional nature of our study does not allow inferences about causality. Also, it is possible that some participants had been previously diagnosed with some of the MetS features, such as hypertension or dyslipidemia, leading to a modification of their diet, which might have weakened the association.

It is known that dietary information from a self-reported FFQ is subject to bias [11]. The possibility of underreporting food consumption by participants who had more MetS features cannot be precluded, for example underreporting of energy intake has been systematically described in overweight and obese individuals [30].

Nevertheless, this study has important strengths such as the relatively large number of participants (2186) and response rate (70%). We assessed dietary intake through a FFQ, which has the advantage of assessing the usual intake, minimizing the effect of the day-to-day variation in food choices.

Also, using the finite mixture models for the identification of DPs have some established advantages [23]. It allows that problems in determining the number of clusters and choosing an appropriate clustering method to be recast as statistical model choice problems. It also produces *posterior* cluster belonging probabilities for each subject, given individual food intakes and any other relevant variables, therefore providing measures of uncertainty of the associated classification (in this study we found high individual posterior probabilities: 89% in women and 94% in men). Additionally, it allows the adjustment of food consumption covariates simultaneously with the fitting process, and allows the size of pattern to depend on a set of (concomitant) variables. Moreover, in the present study, food non-consumption (defined when a person did not eat any food items belonging to a specific food group) was not treated as a continuous variable; our solution included dichotomizing food groups whose non-consumption was, roughly, higher than 50%.

## Conclusions

In this Portuguese sample of non-institutionalized adults, no association was observed between the prevalence of MetS and its individual features and DPs established *a posterior.*

## Abbreviations

MetS: Metabolic Syndrome; DP: Dietary Pattern; BDI: Beck Depression Inventory; FFQ: Food Frequency Questionnaire; BMI: Body Mass Index; WC: Waist Circumference.

## Competing interests

The authors declare that they have no competing interests.

## Authors’ contributions

MJF performed some of the statistical analysis and wrote the first draft of the manuscript. ACS designed the study, participated in the statistical analysis and reviewed the manuscript. RG performed the statistical analysis, regarding the dietary patterns. CL participated in the study design and helped to draft the manuscript. All authors read and approved the final manuscript.
